# A pantothenate suxotroph of BCG rxpressing Gag confers enhanced HIV-specific immunogenicity compared to wildtype and perfingolysin expressing strains

**DOI:** 10.1186/1742-4690-9-S2-P315

**Published:** 2012-09-13

**Authors:** S Chetty, E Shephard, D Bowers, M Darby, W Horsnell, R Chapman, A Williamson

**Affiliations:** 1 IIDMM, University of Cape Town, Cape Town, South Africa; 2 Department of Medicine, University of Cape Town, Cape Town, South Africa

## Background

In tuberculosis vaccine studies, perfingolysin expressing strains (pfo) of recombinant Mycobacterium bovis (rBCG) have been shown to enhance immunogenicity as compared to wildtype strains whilst pantothenate auxotrophic strains (ΔpanCD) have been shown to be safer and more immunogenic. Our group has recently shown that rBCGΔpanCD expressing HIV-1 Gag is more immunogenic than the wildtype Pasteur strain of BCG in the murine model. In this study, a wild type strain, a ΔpanCDstrain, a pfo strain and a ΔpanCD strain expressing perfringolysin (ΔpanCDpfo) of Danish BCG were used as vectors to express HIV-1 subtype C Gag. Gag specific immune responses induced by a prime with each rBCG-Gag vaccine and boost with modified vaccinia Ankara (MVA) were compared.

## Methods

rBCG vaccines were given intraperitoneally to BALB/c mice followed by an intramuscular MVA boost after 28 days. Twelve days after the boost, mice were sacrificed and single cell suspensions of splenocytes were stimulated with HIV peptides. Immunogenicity was assessed using an IFN-γ ELISPOT assay, cytokine bead array and multi-parameter flow cytometry.

## Results

All the rBCG-Gag vaccines primed the immune response to a boost with MVA. Cytokine kinetic measurements and flow cytometry indicated a more rapid and robust release of pro-inflammatory cytokines, in response to ex vivo HIV peptides, from the splenocytes of mice vaccinated with rBCGΔpanCD(gag) as compared to rBCGpfo(gag)(p<0.001- p<0.05) and rBCG(gag)(p<0.01- p<0.05). A rBCGΔpanCD(gag) prime induced increased polyfunctional HIV specific CD4+ and CD8+ T cells as compared to the other strains of BCG. We observed no enhancement of immunogenicity in the rBCGΔpanCDpfo(gag) group as compared to the rBCGΔpanCD(gag) group.

## Conclusion

As this study indicated the Danish ΔpanCD vector induced a more robust and rapid Gag-specific immune response than the wild type and perfringolysin expressing BCG Danish vectors, the Danish ΔpanCD vector maybe an attractive option for HIV vaccines.

